# Safety of guidewire-based measurement of fractional flow reserve and the index of microvascular resistance using intravenous adenosine in patients with acute or recent myocardial infarction

**DOI:** 10.1016/j.ijcard.2015.09.014

**Published:** 2016-01-01

**Authors:** Nadeem Ahmed, Jamie Layland, David Carrick, Mark C. Petrie, Margaret McEntegart, Hany Eteiba, Stuart Hood, Mitchell Lindsay, Stuart Watkins, Andrew Davie, Ahmed Mahrous, Jaclyn Carberry, Vannesa Teng, Alex McConnachie, Nick Curzen, Keith G. Oldroyd, Colin Berry

**Affiliations:** a Institute of Cardiovascular and Medical Sciences, University of Glasgow, Glasgow G12 8QQ, Scotland, UK; b Department of Cardiology, Golden Jubilee National Hospital, Glasgow G81 4DY, Scotland, UK; c Robertson Centre for Biostatistics, University of Glasgow, Glasgow, Scotland, UK; d University Hospital Southampton Foundation Trust, Southampton, UK; e Faculty of Medicine, University of Southampton, Southampton, UK

**Keywords:** Adenosine, ST-elevation myocardial infarction (STEMI), Non ST-elevation myocardial infarction (NSTEMI), Percutaneous coronary intervention (PCI), Blood pressure (BP), Heart rate (HR)

## Abstract

**Aims:**

Coronary guidewire-based diagnostic assessments with hyperemia may cause iatrogenic complications. We assessed the safety of guidewire-based measurement of coronary physiology, using intravenous adenosine, in patients with an acute coronary syndrome.

**Methods:**

We prospectively enrolled invasively managed STEMI and NSTEMI patients in two simultaneously conducted studies in 6 centers (NCT01764334; NCT02072850). All of the participants underwent a diagnostic coronary guidewire study using intravenous adenosine (140 μg/kg/min) infusion for 1–2 min. The patients were prospectively assessed for the occurrence of serious adverse events (SAEs) and symptoms and invasively measured hemodynamics were also recorded.

**Results:**

648 patients (n = 298 STEMI patients in 1 hospital; mean time to reperfusion 253 min; n = 350 NSTEMI in 6 hospitals; median time to angiography from index chest pain episode 3 (2, 5) days) were included between March 2011 and May 2013. Two NSTEMI patients (0.3% overall) experienced a coronary dissection related to the guidewire. No guidewire dissections occurred in the STEMI patients. Chest symptoms were reported in the majority (86%) of patient's symptoms during the adenosine infusion. No serious adverse events occurred during infusion of adenosine and all of the symptoms resolved after the infusion ceased.

**Conclusions:**

In this multicenter analysis, guidewire-based measurement of FFR and IMR using intravenous adenosine was safe in patients following STEMI or NSTEMI. Self-limiting symptoms were common but not associated with serious adverse events. Finally, coronary dissection in STEMI and NSTEMI patients was noted to be a rare phenomenon.

## 1 Introduction

Coronary guidewire-based sensors can be used in the cardiac catheterization laboratory to provide functional information on coronary artery disease severity and microvascular function. The myocardial fractional flow reserve (FFR) assesses the physiological significance of a coronary stenosis and is expressed as the ratio of maximal blood flow in a stenotic artery to maximal flow if theoretically the artery was unobstructed. FFR-guided management is evidence-based in patients with stable coronary artery disease (DEFER [1], FAME [2], FAME-2 [3]) and has emerging clinical utility for measurement of non-infarct artery disease in patients with recent or acute myocardial infarction (MI) (4), (5). The index of microvascular resistance (IMR) measured in the culprit coronary artery has prognostic importance in patients with acute ST-elevation myocardial infarction (STEMI) (6), (7). However since FFR and IMR measurements involve pharmacological hyperemia and guidewire instrumentation, there are theoretical risks of serious adverse events (SAEs), including ventricular arrhythmias with intravenous adenosine and coronary dissection (both ~ 0.5% incidence) [8].

Intravenous adenosine induces hyperemia through interactions with A2A receptors. However, due to the ubiquitous expression of adenosine receptors, adenosine is also associated with unwanted off-target side-effects. For example, interaction with bronchial A2B receptors can lead to mast cell degranulation and bronchoconstriction [9]. Furthermore, activation of cardiac A1 receptors has a myocardial depressant effect with negative chronotropic and dromotropic effects [10]. It is these unwanted effects of adenosine that have motivated researchers to find other drugs for initiation of hyperemia or develop nonhyperemic indices of stenosis assessment in the catheter laboratory (11), (12).

Intracoronary adenosine may also be used therapeutically for the treatment of no-reflow in STEMI (13), (14), and the role of FFR-guided PCI in STEMI patients with multivessel coronary disease [15] is currently being evaluated in the COMPARE-ACUTE (NCT01399736), COMPLETE (NCT01740479) and PRIMULTI (NCT01960933) clinical trials.

In November 2013 the United States (US) Food and Drug Administration (FDA) issued a safety announcement on the risk of myocardial infarction (MI) and death in patients receiving Adenoscan (adenosine) for stress testing [16] (Supplementary File). This announcement followed from reports in the FDA Adverse Event Reporting System (FAERS) and medical literature of serious adverse events (SAEs) from 1995 to 2013, including 6 cases of MI and 27 cases of death following adenosine administration (typically within 6 h) [16].

We aimed to prospectively assess the safety of guidewire based measurement of coronary physiology using intravenous adenosine amongst patients with acute or recent myocardial infarction (MI). Based on our prior experience with intravenous adenosine in this setting (6), (7), (17), (18), we hypothesized that intravenous adenosine would be safe and well tolerated [19].

## 2 Methods

### 2.1 Study population

We simultaneously conducted two prospective studies involving assessments of coronary physiology in patients with acute or recent MI. The first was a study of the natural history of coronary microvascular function in patients with acute STEMI undergoing emergency PCI (the BHF MR-MI study, NCT02072850) [20]. Two hundred and ninety-eight STEMI patients were enrolled acutely and had IMR measured invasively in the culprit coronary artery with a diagnostic coronary guidewire (PressureWire Certus™, St Jude Medical) during primary or rescue PCI. The protocol did not involve FFR or IMR measurements in the non-infarct arteries. The enrolment period was March 2011–November 2012. Patients diagnosed with an acute STEMI [21] and who were undergoing primary or rescue PCI were eligible to participate. In the second study, three hundred and fifty NSTEMI patients were enrolled in the BHF FAMOUS-NSTEMI study (NCT01764334) (4), (5). Six hospitals in the United Kingdom participated (3 academic and 3 non-academic regional hospitals). The patients in this study underwent urgent invasive management and had an FFR measurement in one or more coronary arteries with at least a single coronary stenosis ≥ 30% severity of the reference vessel diameter by visual assessment. The patients with NSTEMI were enrolled during urgent care and the median time to invasive angiography was 3 days (Table 1) [5].

The exclusion criteria for administration of intravenous adenosine included evidence of 2nd or 3rd degree heart block on the ECG, long QT syndrome, cardiogenic shock, or a history of asthma concurrently treated with bronchodilators [22]. The exclusion criteria for both studies are provided in Supplementary Tables 1 and 2. The study was approved by the UK National Research Ethics Service and all participants provided written informed consent.

### 2.2 Catheter laboratory management

The clinical and catheter laboratory management followed contemporary guidelines for STEMI [21] and NSTEMI (21), (23).

### 2.3 Measurement of FFR and IMR

**Fig. 1:**
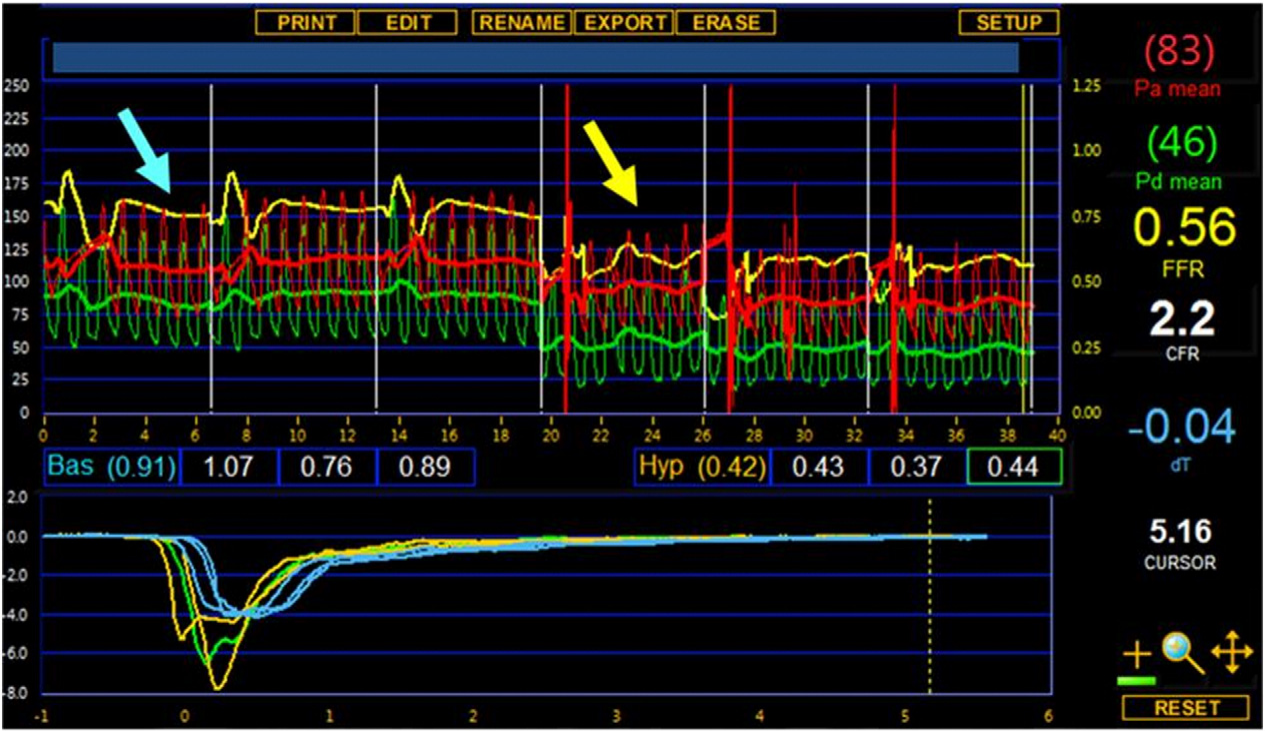
A hemodynamic recording obtained from a diagnostic pressure- and temperature-sensitive guidewire (PressureWire Certus™, St. Jude Medical, Mn.) located in a culprit coronary artery at the end of primary PCI. The blue arrow represents the thermodilution recordings during resting conditions before adenosine administration. The thermodilution curve represents the transit time for the change in temperature detected by the distal guidewire thermistor following intra-coronary bolus injection of saline (room temperature) via the guiding catheter. The subsequent yellow arrow represents the transit times for thermodilution curves following intra-coronary injections of saline during hyperemia with adenosine (140 μg/kg/min). During hyperemia, there is evidence of a reduction in arterial blood pressure depicted by the yellow arrow, reflecting the typical hemodynamic response in the systemic and coronary circulations to intravenous adenosine. Hemodynamic recordings were assessed with vendor software (RADIView Version 2.2, St Jude Medical, Mn.). The systolic blood pressure and diastolic blood pressure during at least 3 cardiac cycles in steady-state conditions at rest and during hyperemia were assessed.

In patients with STEMI, infarct artery microvascular function (IMR) was measured at the end of the primary or rescue PCI (Fig. 1). Thus we initially opted for a conventional workhorse wire while using a pressure wire at the end of the procedure. In patients with NSTEMI, FFR (and IMR) was measured at the beginning of the diagnostic procedure in all participants. Additionally, the pressure wire was used to perform PCI in most NSTEMI patients. FFR and IMR were measured using a temperature and pressure sensitive guide wire (PressureWire Certus™ St Jude Medical, Uppsala, Sweden). The guidewire was calibrated outside the body, equalized with aortic pressure at the ostium of the guide catheter, and then advanced to the distal third of the culprit artery (6), (7), (17), (18). Intracoronary nitrate (200 μg) was administered to minimize coronary artery tone and maintain coronary volume. Intravenous adenosine was administered at a rate of 140 μg/kg/min via a large peripheral vein for 1–2 min (Supplementary Table 3).

The patient's response to adenosine administration was a pre-defined safety outcome [20]. Aortic and distal coronary pressures were recorded invasively before and during adenosine administration. In addition, patients' symptoms and heart rate during the adenosine infusion were also prospectively documented using a study proforma. All SAEs in study participants were prospectively documented by clinical and research staff after the patient was enrolled in the study in line with the trial protocol. All adverse events were recorded in the clinical report form (CRF). SAEs were notified to the Sponsor of the studies for pharmacovigilance and assessed, reported, analyzed and managed in accordance with the Medicines for Human Use (Clinical Trials) Regulations 2004 (as amended) [24].

An SAE was defined as an event that results in death, is life threatening, requires hospitalization or prolongation of existing hospitalization, results in persistent or significant disability or incapacity, or is otherwise considered medically significant by the investigator.

Major adverse cardiac events (MACE), were defined as the occurrence of death, myocardial infarction, or hospitalization for heart failure [25]. In the STEMI study, source data for all of the SAE and MACE were assessed by a cardiologist (A.M.) who was independent of the research team. This cardiologist was blinded to all of the other clinical data [20]. In the NSTEMI study, source clinical data for all SAEs of suspected cardiovascular origin and all deaths were reviewed by an independent clinical event committee blinded to treatment group assignment (FFR-guided group or angiography guided group) (4), (5). The CEC also assessed the angiograms of SAE attributed to procedure-related complications.

### 2.4 Statistics

Continuous data with a normal distribution were summarized with the mean ± standard deviation (SD). Paired t-tests were used to assess hemodynamic data before and during adenosine administration. Significance was defined as a p value < 0.05. The statistical analyses were performed using the SPSS statistical software package 14.0 for Windows (SPSS Inc., Chicago, IL, USA).

## 3 Results

### 3.1 Baseline characteristics

648 patients (n = 298 patients with STEMI in 1 hospital; n = 350 patients with NSTEMI in 6 hospitals) were included between March 2011–May 2013. Their clinical characteristics are presented in Table 1.

In the patients with STEMI, evidence of hemodynamic instability on arrival in the cardiac catheter laboratory was common. Thirty-three (11.1%) patients had a systolic blood pressure (BP) of < 90 mm Hg, 20 (7.2%) patients had ventricular fibrillation (VF) or ventricular tachycardia (VT) before or during PCI but prior to adenosine administration, and 4 (1.4%) patients received intra-aortic balloon pump (IABP) counterpulsation therapy during the PCI. In the patients with NSTEMI there were no patients with VF/VT during the procedure and only 1 (0.3%) patient required IABP.

### 3.2 Symptoms and adverse events

During adenosine infusion, 255 (85.6%) STEMI patients reported symptoms including chest discomfort, dyspnea and facial flushing, all of which resolved immediately after the infusion ceased. There were no other symptoms reported. No MACE, atrial or ventricular arrhythmias occurred in association with intravenous adenosine administration. There were no SAEs related to adenosine.

In the STEMI cohort, MACE occurred in 3 (1.0%) patients within 24 h of the PCI. One patient experienced an acute stent thrombosis associated with a dissection at the distal end of the stent; one patient with severe left ventricular dysfunction experienced ventricular fibrillation in the coronary care unit; one patient died suddenly from myocardial rupture that was confirmed at autopsy. All of these events occurred in the coronary care unit and none of these events were temporally associated with the adenosine infusion in the catheter laboratory. In the STEMI cohort there were no pressure-wire related dissections and no SAE related to arrhythmias.

In the NSTEMI cohort, no MACE occurred in association with the adenosine infusion. There were 2 (0.6%) cases of coronary dissection related to the guidewire. There were 4 cases of in-hospital adverse events, including 3 (0.9%) cases of contrast nephropathy and 3 (0.9%) cases of major bleeding but none related to adenosine infusion. There were no SAE related to bradyarrhythmias or tachyarrhythmias and FFR was measured in all subjects.

### 3.3 Hemodynamic changes

#### 3.3.1 All patients

In 330 patients with complete hemodynamic data (n = 186 STEMI, n = 144 NSTEMI), aortic systolic blood pressure was reduced during adenosine administration (systolic BP (rest vs. adenosine): 124.5 (26.0) mm Hg vs. 111.7 (24.7) mm Hg (n = 330) ([95% CI 12.8 (11.3, 14.3) p < 0.001]) as was diastolic BP (67.0 (12.8) mm Hg vs. 60.5 (13.2) mm Hg (n = 330) ([95% CI 6.5 (5.6, 7.4) p < 0.001]). Heart rate increased to 64.7 (13.0) bpm from 58.3 (12.1) bpm [95% CI 6.3 (5.6, 7.1) p < 0.001]. The proximal aortic pressure (Pa) was also reduced during adenosine administration (systolic BP (rest vs. adenosine): 119.7 (26.6) mm Hg vs. 104.2 (25.0) mm Hg (n = 351) ([95% CI 15.5 (13.9, 17.0) p < 0.001]) as was the distal coronary pressure (64.7 (14.4) mm Hg vs. 55.2 (14.4) mm Hg ([95% CI 9.4 (8.5, 10.4) p < 0.001]).

#### 3.3.2 STEMI

In the STEMI cohort (n = 298), non-invasive hemodynamic data (blood pressure and heart rate) were available for all study participants (Table 2). Complete aortic hemodynamic data before and during adenosine infusion were available in 186 STEMI patients with distal coronary (Pd) hemodynamic data recorded in 258 STEMI patients (Table 3). The mean (SD) aortic systolic BP fell from 120.0 (22.6) mm Hg at baseline to 106.5 (21.3) mm Hg during adenosine infusion [95% CI 13.5 (11.6, 15.5) p < 0.001]. Aortic diastolic BP was also reduced by adenosine infusion (67.9 (13.5) mm Hg vs. 61.0 (13.6) mm Hg) [95% CI 7.0 (5.8, 8.1) p < 0.001] whereas heart rate increased from 63.2 (12.1) bpm at rest to 69.8 (12.5) bpm [95% CI 6.6 (5.6, 7.6) p < 0.001]. Compared to patients who did not experience symptoms with adenosine, patients who did experience symptoms had a greater rise in heart rate, but BP changes were similar (Table 4).

#### 3.3.3 NSTEMI

In the NSTEMI cohort (n = 350), complete aortic hemodynamic data were available for 144 NSTEMI patients with distal coronary (Pd) hemodynamic data recorded in 165 NSTEMI patients. The mean (SD) non-invasive aortic systolic BP reduced from 130.3 (28.8) mm Hg under resting conditions to 118.5 (27.0) mm Hg during adenosine infusion [95% CI 11.8 (9.4, 14.2) p < 0.001]. Aortic diastolic BP was also reduced by adenosine infusion (65.9 (11.9) mm Hg vs. 60.0 (12.7) mm Hg [95% CI 5.9 (4.5, 7.2) p < 0.001]). Heart rate increased to 58.1 (11.0) bpm from 52.1 (8.8) bpm (n = 144) [95% CI 6.0 (4.8, 7.2) p < 0.001] and distal coronary (Pd) pressure was reduced also (Table 5).

## 4 Discussion

We report the largest study to date of guidewire-based measurements of FFR and/or IMR in patients with acute coronary syndromes. Our study is the first to report information on a pre-specified outcome relating to the safety of intravenous adenosine in patients with an acute STEMI or recent NSTEMI, who were prospectively enrolled simultaneously in parallel studies. The main findings of our multicenter study are that, first, coronary dissection due to the guidewire was rare (≤ 0.3%). Second, brief intravenous adenosine infusion in MI patients for diagnostic purposes was commonly associated with symptoms but these symptoms were brief and self-limiting and were not associated with any SAEs; most importantly, the use of adenosine was safe and not associated with any SAEs during routine emergency care.

Only 2 guidewire-related coronary dissections occurred in 698 prospectively enrolled MI patients undergoing emergency or urgent invasive management. This result represents evidence for the safety of guidewire-based assessments of coronary physiology. Guidewire dissections were less common than in previous studies [e.g., RIPCORD (1.5%)] [8]. We believe that the timing of the pressure wire study within the procedure partially explains the difference in the dissection rates. In the patients with acute STEMI, pressure wire instrumentation in the infarct-artery post-PCI was not associated with any complications. In the patients with recent NSTEMI, the 2 guidewire dissections occurred during diagnostic procedures before stent implantation. In the 350 NSTEMI participants in this trial, 706 lesions (≥ 30% lumen narrowing) were reported and FFR data were obtained in 704 (> 99%) of these lesions. On average 2 arteries per patient were instrumented with a pressure wire. Despite this, the incidence of guidewire dissections in the NSTEMI patients was very low and this experience is evidence of safety in the hands of trained cardiologists.

In our study, predictable symptoms associated with intravenous adenosine occurred in the majority of patients and can be explained by the pharmacological effects of this naturally occurring vasodilator [25]. However, since the half life of adenosine is < 10 s, these symptoms were extremely short-lived [26]. Patients who experienced symptoms had a slightly higher increase in heart rate. A minority of patients (14%) did not experience symptoms with adenosine infusion. This may be explained by the presence of concurrent chest symptoms associated with myocardial infarction and also treatment with sedative and opiate therapies. There were no serious adverse events associated with intravenous adenosine. None of the patients experienced sustained atrial or ventricular tachyarrhythmia. Overall, the reported symptoms that were observed in these cohorts were typical of what might be expected with intravenous adenosine. Based on the evidence of safety in this study, we think that when a clinician plans to administer intravenous adenosine, the patient should be advised that symptoms are likely but self-limiting and not associated with any other consequences. Adverse events, such as atrial and ventricular fibrillation, are rare [8] and, in fact, no such events occurred in the 648 MI patients in this analysis.

Systolic and diastolic blood pressures were reduced with intravenous adenosine consistent with an A2A receptor-mediated response. However, a rise in heart rate of > 10% or a fall in systolic BP > 10% occurred in less than half of the STEMI and NSTEMI patients in our study. These observations could be explained by the fact that the patients already had tachycardia due to STEMI, and they had been treated with vasoactive drugs, which attenuate the systemic reflex sympatho-excitation response (e.g., intravenous morphine). It is also possible that there was an attenuated sympathetic response due to beta blocker treatment.

Of the 6 hospitals in the FAMOUS-NSTEMI trial (4), (5), 3 were regional non-academic centers without a track record in coronary physiology research. The multicenter design was intended to make the results of this trial more representative of routine care, relevant to “real world” practice and novel.

In STEMI patients, guidewire-based measurement of coronary microvascular function with intravenous adenosine is mainly used in clinical research studies (as was the case here in the STEMI group). IMR in the infarct-related artery has prognostic value when measured invasively at the end of primary PCI (6), (7), (17), (18), and IMR has potential utility for stratification of higher risk patients for more intensive management after primary PCI (6), (18). Moreover, potential diagnostic applications are emerging for FFR to inform the acute treatment decisions for patients with non-infarct artery disease [5]. Intracoronary adenosine is used to treat no-reflow (13), (14) and FFR-guided PCI in STEMI patients with multivessel coronary disease [15], is currently being evaluated in the COMPARE-ACUTE (NCT01399736), COMPLETE (NCT01740479) and PRIMULTI (NCT01960933) trials.

In our study invasive measurements of FFR and IMR can be safely obtained in patients with acute or recent MI. IMR has been shown to be a biomarker of severe microvascular injury and has prognostic value in identifying the highest risk patients at an early stage, potentially enabling triage of higher risk patients to intensification of therapy. FFR measurement in the acute setting, especially in non-culprit disease, has much promise and is undergoing ongoing evaluation in clinical trials.

Adenosine is an established drug for use in pharmacological stress testing. Adenoscan has been marketed from 18 May 1995. From this date until 10 April 10 2013 the FAERS database accrued 26 reports of myocardial infarction (MI) and 29 deaths with regadenoson and 6 reports of myocardial infarction and 27 deaths reported with Adenoscan. There were two case reports of MI associated with Lexiscan administration but none with Adenoscan (27), (28) and the incidence of cardiovascular adverse events associated with these drugs is similarly uncommon (29), (30), (31), (32), (33). In light of these post-marketing reports the FDA recommended to “*Avoid using these drugs* (*Lexiscan or Adenoscan*) *in patients with signs or symptoms of unstable angina or cardiovascular instability*, *as these patients may be at greater risk for serious cardiovascular adverse reactions*” [16]. The FDA warning was directed to office-based administration of intravenous adenosine, and this environment contrasts with the cardiac catheterization laboratory where medical support is immediately available to treat patients with iatrogenic complications. Our results also provide reassurance for the use of intravenous adenosine in the catheter laboratory setting.

In contrast to the FDA recommendations, our findings are supported by the results from similar studies in other centers, in which intravenous adenosine has been used in patients with acute MI (7), (34), (35), (36), (37). Moreover, a meta-analysis evaluating the safety and efficacy of intracoronary adenosine in 460 patients with STEMI undergoing PCI found no difference in the safety endpoints of bradycardia, ventricular arrhythmia and chest pain compared with placebo [38]. Our study is different, since the safety of diagnostic guidewire instrumentation and systemic administration of adenosine (rather than intracoronary adenosine) were prospectively assessed in NSTEMI and STEMI patients. Another study, using a similar protocol for adenosine, demonstrated all patients tolerated adenosine infusion, with no episodes of clinically significant bradycardia [34]. In our hands, adenosine was not associated with any SAE when administered to reperfused patients with STEMI at the end of emergency PCI for a short period of time (1–2 min) and the absence of SAE in the NSTEMI patients provides further evidence of safety.

## 5 Limitations

Our study has several limitations. First, we do not have information on other hyperemic drugs, such as regadenoson. Second, although safety assessments were performed and recorded in all of the patients at the time of the procedure, symptom reporting was incomplete. The available results confirm that symptoms typically occur with intravenous adenosine. Third, complete hemodynamic recordings were not available in all of the participants because resting arterial pressure is not required for IMR. Fourth, pressure wire studies were restricted to the infarct-related artery rather than the non-infarct artery in the STEMI cohort study. Nonetheless, we provide comprehensive hemodynamic data and information on symptoms from prospective evaluations in individual patients who were enrolled in studies that had been designed with an open approach to enrolment of ‘all-comers’. We think our observations are representative of ‘real-world’ clinical practice.

## 6 Conclusion

Guidewire-based measurement of coronary microvascular function involving intravenous adenosine infusion was feasible and safe during emergency or urgent PCI for STEMI and NSTEMI. The symptoms related to adenosine were predictable, self-limiting and not associated with adverse events. Finally, coronary dissection in MI patients was noted to be a rare phenomenon.

## Funding sources

Our research is supported by grants from the British Heart Foundation (BHF) (Project Grant PG/11/2/28474), Chief Scientist Office (CSO), Scottish Diabetes Research Network (SDRN), Medical Research Scotland and the Scottish Funding Council (SFC).

## Conflicts of interest statement

All authors participating have disclosed potential conflicts of interest. Professor Berry has been Principal Investigator on institutional research grants supported by St Jude Medical and he has acted as a consultant to St Jude Medical with reimbursement to his employer, the University of Glasgow. Professor Oldroyd and Professor Curzen have acted as consultants to Volcano Corporation and St Jude Medical. None of the other authors have conflicts of interest to declare.

## Figures and Tables

**Table 1:** Clinical characteristics of the study participants on admission.

Characteristics		STEMI patients n = 298	NSTEMI patients n = 350
*Clinical*			
Age, years		59.4	62.0
Male sex, n (%)		216 (72)	260 (74)
BMI (kg/m^2^)		28.7	29 (5)

*History*			
Hypertension, n (%)		95 (32)	159 (45)
Current smoking, n (%)		184 (62)	143 (41)
Hypercholesterolemia, n (%)		81 (27)	127 (36)
Diabetes mellitus ‡, n (%)		32 (11)	52 (15)
Previous myocardial infarction, n (%)		20 (7)	46 (13)
Previous PCI, n (%)		16 (5)	38 (11)

*Presenting characteristics*			
Heart rate, bpm		80 (44)	74 (16)
Systolic blood pressure, mm Hg		135 (25)	141 (27)
Diastolic blood pressure, mm Hg		79 (14)	81 (17)
Time from symptom onset to reperfusion, min		253	–
Time from index episode of myocardial ischemia to invasive angiogram, days		–	3 (2, 5)
Ventricular tachycardia or fibrillation †, n (%)		20 (7)	0 (0)
Heart failure, Killip class at presentation, n (%)	I	212 (71)	308 (88)
II	64 (22)	33 (9)
III	16 (5)	5 (2)
IV	6 (2)	4 (1)

*Coronary angiography*			
Reperfusion strategy, n (%)			
Primary PCI		275 (92)	–
Rescue PCI (failed thrombolysis)		23 (8)	–
Adjunctive therapy during PCI			
Aspirin (%)		297 (99)	348 (99)
Clopidogrel (600 mg) (%)		297 (99)	337 (96)
Heparin (%)		298 (100)	333 (95)
Anti-GP IIb/IIIa (%)		273 (92)	79 (26)
Number of diseased arteries, n (%)	0	0 (0)	10 (3)
1	165 (55)	130 (37)
2	95 (32)	141 (40)
3	38 (13)	60 (17)
4	0 (0)	9 (3)
Culprit artery, n (%)	LMS	0 (0)	2 (1)
LAD	110 (37)	152 (43)
LCX	55 (18)	106 (30)
RCA	133 (45)	90 (26)
TIMI coronary flow grade pre-PCI, n (%)	0/1	214 (72)	–
2	56 (19)	–
3	28 (9)	–
TIMI coronary flow grade post-PCI, n (%)	0/1	2 (1)	33 (9)
2	14 (5)	27 (8)
3	282 (94)	289 (83)

Abbreviations: body mass index (BMI), percutaneous coronary intervention (PCI), beats per minute (bpm), thrombolysis in myocardial infarction (TIMI), left main stem (LMS), left anterior descending (LAD), left circumflex (LCX), right coronary artery (RCA).

‡ Diabetes mellitus was defined as a history of diet-controlled or treated diabetes.

† VF or VT before or during PCI, but prior to adenosine infusion.

**Table 2:** Blood pressure and heart rate at the start and end of emergency PCI in 298 STEMI subjects.

Parameter	PCI start	PCI end	Mean Change (CI)	p value
Mean heart rate (SD) bpm	80.1 (44.1)	79.5 (14.5)	0.6 (− 4.3, 5.6)	0.800
Mean systolic BP (SD) mm Hg	135.1 (24.7)	121.0 (21.1)*	14.0 (11.6, 16.4)	< 0.001
Mean diastolic BP (SD) mm Hg	79.0 (13.9)	71.9 (12.9)*	7.0 (5.6, 8.5)	< 0.001

Abbreviations: heart rate (HR), blood pressure (BP), standard deviation (SD), *p < 0.001 vs. baseline (paired t-test).

**Table 3:** Coronary (Pd) systolic and diastolic blood pressure recorded in 258 STEMI subjects.

Blood pressure (BP)	Rest	Adenosine	Mean change (CI)	p value
Mean systolic (SD) mm Hg	114.6 (22.6)	98.8 (21.8)*	15.8 (14.1, 17.5)	< 0.001
Mean diastolic (SD) mm Hg	65.5 (13.9)	56.1 (14.4)*	9.4 (8.4, 10.3)	< 0.001

Abbreviations: blood pressure (BP), standard deviation (SD), *p < 0.001 vs. baseline (paired t-test).

**Table 4:** Blood pressure and heart rate of STEMI patients with symptoms recorded (n = 186) and who reported symptoms vs. no symptoms.

Parameter	Symptoms (n = 154)	No symptoms (n = 32)	Mean difference (CI)	p value
Mean (SD) systolic change, mm Hg	− 14.2 (13.5)	− 10.5 (13.5)	− 3.6 (− 8.3, 1.5)	0.167
Mean (SD) diastolic change, mm Hg	− 7.2 (7.8)	− 6.0 (8.4)	− 1.2 (− 4.2, 1.9)	0.451
Mean (SD) HR change, bpm	7.1 (7.0)	4.0 (6.2)*	3.1 (0.5, 5.7)	0.020

Abbreviations: heart rate (HR), standard deviation (SD), *p < 0.05 vs. baseline (unpaired t-test).

**Table 5:** Distal coronary (Pd) artery blood pressure recorded in 165 NSTEMI subjects.

Blood pressure (BP)	Rest	Adenosine	Mean change (CI)	p value
Mean systolic (SD) mm Hg	125.7 (28.3)	110.4 (26.4)*	15.3 (13.0, 17.7)	< 0.001
Mean diastolic (SD) mm Hg	63.7 (14.2)	53.7 (13.7)*	10.0 (8.4, 11.5)	< 0.001

Abbreviations: blood pressure (BP), standard deviation (SD), *p < 0.001 vs. baseline (paired t-test).
