# Supplementary material for: Safety of guidewire-based measurement of fractional flow reserve and the index of microvascular resistance using intravenous adenosine in patients with acute or recent myocardial infarction
Source: Int J Cardiol. 2016 Jan 1;202:305–10. doi: 10.1016/j.ijcard.2015.09.014 (PMC4669307; doi:10.1016/j.ijcard.2015.09.014)
Supplement: Supplementary file 1 — Supplementary material. [file mmc1.doc]

# Supplementary File

**Table 1.** Exclusion criteria for participation in the STEMI cohort study [1].

| Exclusion Criteria |
| --- |
| 2^nd^ or 3^rd^ degree heart block on ECG |
| Long QT syndrome |
| Cardiogenic shock |
| History of asthma concurrently treated with bronchodilators |
| Terminal systemic illness (e.g. cancer limiting survival <12 months) |
| Pregnancy |
| Inability to provide informed consent |

Abbreviations – Electrocardiogram (ECG), ST-elevation myocardial infarction (STEMI)

**Table 2.** Exclusion criteria for participation in the FAMOUS-NSTEMI clinical trial [2][3].

| NSTEMI Exclusion Criteria |
| --- |
|  |
|  |
|  |
|  |
|  |
|  |
|  |
|  |
|  |
|  |
|  |
|  |
|  |
|  |
|  |
|  |
|  |
|  |
|  |
|  |
|  |
|  |
|  |
|  |
|  |
| Contra-indications to intravenous adenosine including 1) A history of asthma concurrently treated with bronchodilators; 2) 2^nd^ or 3^rd^ degree heart block on ECG; 3) Long QT syndrome |
| A life expectancy < 1 year (e.g. cancer limiting survival <12 months) |
| Pregnancy |
| Inability to provide informed consent |
| Hemodynamic Instability |
| MI with persistent ST elevation |
| Ineligible for coronary revascularization on clinical grounds |
| Plan for non-coronary heart surgery  Prior CABG  Angiographic evidence of severe (e.g. diffuse calcification) or mild (< 30% severity) coronary disease  Intolerance to anti-platelet drugs, |

Abbreviations – Electrocardiogram (ECG), Myocardial Infarction (MI), Non-ST elevation myocardial infarction (NSTEMI).

**Table 3.** Preparation of intravenous adenosine.

| Step | Action |
| --- | --- |
| 1. | Remove 40ml from a 100ml bag of IV saline and discard |
| 2. | Draw up 30ml (90mg) of adenosine (3mg/ml either 15x2ml vials or 3x10ml vials) |
| 3. | Add adenosine to the 60 ml IV saline giving a concentration of 1mg/ml |

Abbreviations – Intravenous (IV), millilitres (ml), milligram (mg)

#### FDA **Safety Announcement [20 November 2013]**

*"The U.S. Food and Drug Administration (FDA) is warning health care professionals of the rare but serious risk of heart attack and death with use of the cardiac nuclear stress test agents Lexiscan (regadenoson) and Adenoscan (adenosine) [4].  We have approved changes to the drug labels to reflect these serious events and updated our recommendations for use of these agents [4].  Health care professionals should avoid using these drugs in patients with signs or symptoms of unstable angina or cardiovascular instability, as these patients may be at greater risk for serious cardiovascular adverse reactions [4].*

*Lexiscan and Adenoscan are FDA approved for use during cardiac nuclear stress tests in patients who cannot exercise adequately. Lexiscan and Adenoscan help identify coronary artery disease. They do this by dilating the arteries of the heart and increasing blood flow to help identify blocks or obstructions in the heart’s arteries. Lexiscan and Adenoscan cause blood to flow preferentially to the healthier, unblocked or unobstructed arteries, which can reduce blood flow in the obstructed artery. In some cases, this reduced blood flow can lead to a heart attack, which can be fatal.*

*The* Warnings & Precautions *section of the Lexiscan and Adenoscan labels previously contained information about the possible risk of heart attack and death with use of these drugs.  However, recent reports of serious adverse events in the FDA Adverse Event Reporting System (FAERS) database and the medical literature [5] [6] prompted us to approve changes to the drug labels to include updated recommendations for use. Some events occurred in patients with signs or symptoms of acute myocardial ischemia, such as unstable angina or cardiovascular instability.  Cardiac resuscitation equipment and trained staff should be available before administering Lexiscan or Adenoscan.  At this time, data limitations prevent us from determining if there is a difference in risk of heart attack or death between Lexiscan and Adenoscan. We recommend that health care professionals and their patients discuss any questions or concerns."*

*FDA reviewed the FDA Adverse Event Reporting System (FAERS) database and the medical literature for cases of myocardial infarction (MI) and death from all causes associated with Lexiscan (regadenoson) and Adenoscan (adenosine).  We analyzed FAERS data for Lexiscan from June 24, 2008, to April 10, 2013, and for Adenoscan, from May 18, 1995, to April 10, 2013. The beginning dates correlated with the start of marketing for each drug.*

*We identified cases of MI and deaths from all causes for both Lexiscan and Adenoscan. The FAERS database contained 26 MI cases and 29 cases of death occurring after Lexiscan administration, and six cases of MI and 27 cases of death following Adenoscan administration. Reports did not always specify when deaths or MIs occurred.  When reported, these adverse events tended to occur within 6 hours following Lexican or Adenoscan administration.  A few deaths occurred when Lexiscan or Adenoscan was administered with exercise stress testing, which is not an FDA approved use of the drugs.*

*With Lexiscan, the most common adverse events associated with death were cardiac arrest, MI, loss of consciousness, respiratory arrest, electrocardiogram ST segment depression, pulmonary edema, and ventricular fibrillation.  With Adenoscan, the most common adverse events associated with death were cardiorespiratory arrest, dyspnea, cardiac arrest, respiratory arrest, and ventricular tachycardia.*

*The number of postmarketing reports is subject to change over time, and may not reflect the true proportion of cases associated with either Lexiscan or Adenoscan.  Many factors can influence whether adverse effects are reported, particularly the length of time a drug has been marketed, whether or not the adverse effect is described in the drug label, and the amount of publicity about an event or safety concern.  Specifically for Lexiscan and Adenoscan, the analysis is complicated by differences in the number of patient exposures and in underlying cardiac risk factors that can influence choice of drug, and for Adenoscan, by its longer time on the market.*

Medical Literature Review

*A review of the medical literature also identified two case reports of MI associated with Lexiscan [5] [6]. However, published studies from the medical literature have not documented an increased incidence of cardiovascular adverse events with Lexiscan compared to Adenoscan [7] [8] [9] [10] [11].*

#### **References**

1. Berry C. The British Heart Foundation Magnetic Resonance imaging in Myocardial Infarction (MR-MI) Study. <http://clinicaltrials.gov/ct2/show/NCT02072850>
2. Berry C, Layland J, Sood A, Curzen NP, Balachandran KP, Das R, Junejo S, Henderson RA, Briggs AH, Ford I, Oldroyd KG. Fractional flow reserve versus angiography in guiding management to optimize outcomes in non-ST-elevation myocardial infarction (FAMOUS-NSTEMI): rationale and design of a randomized controlled clinical trial. Am Heart J 2013;166:662–668.
3. Layland J, Oldroyd KG, Curzen N, Sood A, Balachandran K, Das R, Juneju S, Ahmed N, Lee M, Shaukat A, O’Donnell A, Nam J, Briggs A, Henderson R, McConnachie A, Berry C. Fractional flow reserve versus angiography in guiding management to optimise outcomes in non-ST-segment elevation myocardial infarction: the British Heart Foundation FAMOUS-NSTEMI randomized trial. Eur Heart J 2014; doi:10.1093/eurheartj/ehu338.
4. FDA warns of rare but serious risk of heart attack and death with cardiac nuclear stress test drugs Lexiscan (regadenoson) and Adenoscan (adenosine) <http://www.fda.gov/drugs/drugsafety/ucm375654.htm Safety Announcement. 11-20-2013>
5. Shah S, Parra D, Rosenstein RS. Acute myocardial infarction during regadenoson myocardial perfusion imaging. Pharmacotherapy 2013;33:90-5.
6. Hsi DH, Marreddy R, Moshiyakhov M, Luft U. Regadenoson induced acute ST-segment elevation myocardial infarction and multivessel coronary thrombosis. J Nucl Cardiol 2013;20:481-4.
7. Iskandrian AE, Bateman TM, Belardinelli L, Blackburn B, Cerqueira MD, Hendel RC, [Lieu H](http://www.ncbi.nlm.nih.gov/pubmed/?term=Lieu H%5BAuthor%5D&cauthor=true&cauthor_uid=17826318), [Mahmarian JJ](http://www.ncbi.nlm.nih.gov/pubmed/?term=Mahmarian JJ%5BAuthor%5D&cauthor=true&cauthor_uid=17826318), [Olmsted A](http://www.ncbi.nlm.nih.gov/pubmed/?term=Olmsted A%5BAuthor%5D&cauthor=true&cauthor_uid=17826318), [Underwood SR](http://www.ncbi.nlm.nih.gov/pubmed/?term=Underwood SR%5BAuthor%5D&cauthor=true&cauthor_uid=17826318), [Vitola J](http://www.ncbi.nlm.nih.gov/pubmed/?term=Vitola J%5BAuthor%5D&cauthor=true&cauthor_uid=17826318), [Wang W](http://www.ncbi.nlm.nih.gov/pubmed/?term=Wang W%5BAuthor%5D&cauthor=true&cauthor_uid=17826318); [ADVANCE MPI Investigators](http://www.ncbi.nlm.nih.gov/pubmed/?term=ADVANCE MPI Investigators%5BCorporate Author%5D). Adenosine versus regadenoson comparative evaluation in myocardial perfusion imaging: results of the ADVANCE phase 3 multicenter international trial. J Nucl Cardiol 2007;14:645-58.
8. Thomas GS, Thompson RC, Miyamoto MI, Ip TK, Rice DL, Milikien D, [Lieu HD](http://www.ncbi.nlm.nih.gov/pubmed/?term=Lieu HD%5BAuthor%5D&cauthor=true&cauthor_uid=19152130), [Mathur VS](http://www.ncbi.nlm.nih.gov/pubmed/?term=Mathur VS%5BAuthor%5D&cauthor=true&cauthor_uid=19152130). The RegEx trial: a randomized, double-blind, placebo- and active-controlled pilot study combining regadenoson, a selective A(2A) adenosine agonist, with low-level exercise, in patients undergoing myocardial perfusion imaging. J Nucl Cardiol 2009;16:63-72.
9. Cavalcante JL, Barboza J, Ananthasubramaniam K. Regadenoson is a safe and well-tolerated pharmacological stress agent for myocardial perfusion imaging in post-heart transplant patients. J Nucl Cardiol 2011;18:628-33.
10. Nair PK, Marroquin OC, Mulukutla SR, Khandhar S, Gulati V, Schindler JT, Lee JS. Clinical utility of regadenoson for assessing fractional flow reserve. JACC Cardiovasc Interv 2011;4:1085-92.
11. Arumugham P, Figueredo VM, Patel PB, Morris DL. Comparison of intravenous adenosine and intravenous regadenoson for the measurement of pressure-derived coronary fractional flow reserve. Euro Intervention 2013;8:1166-71.
